# (*E*)-2-{4-[1-(Hydroxyimino)ethyl]phenyl­iminomethyl}-6-methoxyphenol mono­hydrate

**DOI:** 10.1107/S1600536809042032

**Published:** 2009-10-17

**Authors:** Jun-Feng Tong, Su-Xia Gao, Wen-Kui Dong, Hong-Fu Li, Jian-Chao Wu

**Affiliations:** a School of Chemical and Biological Engineering, Lanzhou Jiaotong University, Lanzhou 730070, People’s Republic of China; b School of Environmental Science and Municipal Engineering, Lanzhou Jiaotong University, Lanzhou 730070, People’s Republic of China

## Abstract

In the title compound, C_16_H_16_N_2_O_3_·H_2_O, the benzene rings are nearly coplanar with each other, forming a dihedral angle of 4.46 (3)°. There is a strong intra­molecular O—H⋯N hydrogen bond which results in a six-membered ring. In the crystal, the mol­ecules are connected into a three-dimensional network *via* O—H⋯O and O—H⋯N inter­molecular hydrogen bonds, forming a centrosymmetric ring along the *b* axis with graph-set motif *R*_4_^4^(10). In addition, the short distances between the centroids of six-membered rings [3.555 (1) Å], indicate the existence of π–π stacking inter­actions, which may stabilize the crystal structure.

## Related literature

For background to oximes, see: Chaudhuri (2003 ▶); Dong *et al.* (2008 ▶, 2009 ▶); Zhao *et al.* (2009 ▶). For bond-length data, see: Allen *et al.* (1987 ▶). For graph-set analysis of hydrogen bonding, see: Bernstein *et al.* (1995 ▶).
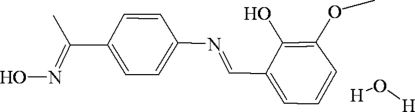


## Experimental

### 

#### Crystal data

C_16_H_16_N_2_O_3_·H_2_O
*M* *_r_* = 302.32
Triclinic, 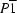

*a* = 8.1030 (14) Å
*b* = 8.3273 (15) Å
*c* = 12.4392 (16) Å
α = 72.095 (1)°
β = 80.012 (2)°
γ = 69.454 (1)°
*V* = 745.9 (2) Å^3^
*Z* = 2
Mo *K*α radiation
μ = 0.10 mm^−1^
*T* = 298 K
0.45 × 0.33 × 0.13 mm

#### Data collection

Bruker SMART 1000 CCD area detector diffractometer
Absorption correction: multi-scan (*SADABS*; Sheldrick, 1996 ▶) *T*_min_ = 0.957, *T*_max_ = 0.987
3912 measured reflections
2586 independent reflections
1202 reflections with *I* > 2σ(*I*)
*R*_int_ = 0.032

#### Refinement

*R*[*F* ^2^ > 2σ(*F* ^2^)] = 0.056
*wR*(*F* ^2^) = 0.149
*S* = 1.02
2586 reflections
200 parameters
H-atom parameters constrained
Δρ_max_ = 0.24 e Å^−3^
Δρ_min_ = −0.22 e Å^−3^

### 

Data collection: *SMART* (Siemens, 1996 ▶); cell refinement: *SAINT* (Siemens, 1996 ▶); data reduction: *SAINT*; program(s) used to solve structure: *SHELXS97* (Sheldrick, 2008 ▶); program(s) used to refine structure: *SHELXL97* (Sheldrick, 2008 ▶); molecular graphics: *SHELXTL* (Sheldrick, 2008 ▶); software used to prepare material for publication: *SHELXTL*.

## Supplementary Material

Crystal structure: contains datablocks global, I. DOI: 10.1107/S1600536809042032/pv2215sup1.cif

Structure factors: contains datablocks I. DOI: 10.1107/S1600536809042032/pv2215Isup2.hkl

Additional supplementary materials:  crystallographic information; 3D view; checkCIF report

## Figures and Tables

**Table 1:** Hydrogen-bond geometry (Å, °)

*D*—H⋯*A*	*D*—H	H⋯*A*	*D*⋯*A*	*D*—H⋯*A*
O1—H1⋯O4^i^	0.82	1.84	2.656 (3)	176
O2—H2⋯N2	0.82	1.86	2.589 (3)	147
O4—H4*A*⋯O2^ii^	0.85	2.07	2.885 (3)	161
O4—H4*B*⋯N1^iii^	0.85	2.15	2.945 (3)	156

Symmetry codes: (i) 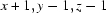; (ii) 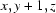; (iii) 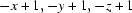.

**Fig. 1.**
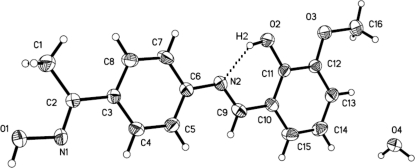
The molecule structure of the title compound with the atom numbering scheme. Displacement ellipsoids for non-hydrogen atoms are drawn at the 30% probability level.

**Fig. 2.**
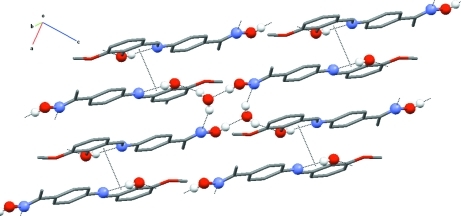
Packing interactions in the title compound, showing the intra- and intermolecular hydrogen bonds as well as π···π stacking; H atoms not involved in hydrogen bonding have been omitted for clarity.

## supplementary crystallographic information

### Comment

Oximes are a classical type of chelating ligands which are widely used in coordination and analytical chemistry (Chaudhuri, 2003). In continuation of our study (Zhao *et al.*, 2009; Dong *et al.*, 2008; Dong *et al.*, 2009) on oxime-type compounds, herein, we report the synthesis and crystal structure of the title compound (I).

The asymmetric unit of the title compound (Fig. 1), which is a potential bidentate oxime-type ligand, contains one (*E*)-4-[1-(hydroxyimino)ethyl]-*N*-(2-hydroxy-3-methoxybenzylidene)aniline and one water molecule. The bond lengths and angles in the molecule are within normal ranges (Allen *et al.*, 1987). Two benzene rings (C3—C8 and C10—C15) are nearly coplanar with each other, making a dihedral angle of 4.46 (3)°. The torsion angles O1—N1—C2—C3 and C6—N2—C9—C10 are -178.7 (2) and -178.9 (2)°, respectively.

In the title compound, a strong intramolecular O—H···N hydrogen bond forms a six-membered ring, producing an S(6) ring motif (Bernstein *et al.*, 1995). The molecules of (I) are connected into a three-dimensional hydrogen-bonded network *via* O—H···O and O—H···N hydrogen bonds, thus generating double layers, the junction between them is ensured by intermolecular O4—H4B···N1, O1—H1···O4 hydrogen bonds which can be described by the graph-set motif of *R*_4_^4^(10) (Bernstein *et al.*, 1995) and O4—H4A···O2 hydrogen bonds (Table 1, Fig. 2) *via* a water molecule (H_2_O, (O4)), forming a centrosymmetric ring along the *b* axis. In addition, short distances between the centroids of six-membered rings [3.555 (1) Å], shows the existence of π···π stacking interactions which may stabilize the crystal structure (Fig. 2).

### Experimental

To a pale-yellow solution of  2-hydroxy-3-methoxybenzaldehyde (152.2 mg, 1.00 mmol) in ethanol (3 ml) was added a colorless solution of 1-(*p*-aminophenyl)ethanone oxime (143.5 mg, 0.96 mmol) in ethanol (3 ml). The mixture was stirred at 328–333 K for 13 h. On cooling the mixture to room temperature, a red precipitate was formed which was filtered under reduced pressure and washed successively with ethanol (2 ml) and n-hexane (6 ml). The product was dried under vacuum and purified with recrystallization from ethanol to yield the title compound. Red block-like single crystals suitable for X-ray diffraction studies were obtained after two weeks by slow evaporation from an acetone solution of the title compound at room temperature.

### Refinement

H atoms were treated as riding atoms with distances C—H = 0.96 Å (CH_3_), 0.93 Å (CH), O—H = 0.82 Å for (OH) and 0.85 Å (H_2_O). The isotropic displacement parameters for all H atoms were set equal to 1.2 or 1.5 *U*_eq_ of the carrier atom.

### Crystal data

**Table:**

C_16_H_16_N_2_O_3_·H_2_O	*Z* = 2
*M**_r_* = 302.32	*F*(000) = 320
Triclinic, *P*1	*D*_x_ = 1.346 Mg m^−^^3^
Hall symbol: -P 1	Melting point = 462–464 K
*a* = 8.1030 (14) Å	Mo *K*α radiation, λ = 0.71073 Å
*b* = 8.3273 (15) Å	Cell parameters from 714 reflections
*c* = 12.4392 (16) Å	θ = 2.7–24.1°
α = 72.095 (1)°	µ = 0.10 mm^−^^1^
β = 80.012 (2)°	*T* = 298 K
γ = 69.454 (1)°	Block-like, red
*V* = 745.9 (2) Å^3^	0.45 × 0.33 × 0.13 mm

### Data collection

**Table:**

Bruker SMART 1000 CCD area detector diffractometer	2586 independent reflections
Radiation source: fine-focus sealed tube	1202 reflections with *I* > 2σ(*I*)
graphite	*R*_int_ = 0.032
φ & ω scans	θ_max_ = 25.0°, θ_min_ = 1.7°
Absorption correction: multi-scan (*SADABS*; Sheldrick, 1996)	*h* = −6→9
*T*_min_ = 0.957, *T*_max_ = 0.987	*k* = −8→9
3912 measured reflections	*l* = −14→14

### Refinement

**Table:**

Refinement on *F*^2^	Primary atom site location: structure-invariant direct methods
Least-squares matrix: full	Secondary atom site location: difference Fourier map
*R*[*F*^2^ > 2σ(*F*^2^)] = 0.056	Hydrogen site location: inferred from neighbouring sites
*wR*(*F*^2^) = 0.149	H-atom parameters constrained
*S* = 1.02	*w* = 1/[σ^2^(*F*_o_^2^) + (0.057*P*)^2^] where *P* = (*F*_o_^2^ + 2*F*_c_^2^)/3
2586 reflections	(Δ/σ)_max_ < 0.001
200 parameters	Δρ_max_ = 0.24 e Å^−^^3^
0 restraints	Δρ_min_ = −0.22 e Å^−^^3^

### Special details

**Table:**

Experimental. Yield (164.9 mg) 60.69%. m. p. 462–464 K. Anal. Calcd. for C_16_H_18_N_2_O_4_: C, 63.56; H, 6.00; N, 9.27. Found: C, 63.40; H, 5.89; N, 9.35.
Geometry. All e.s.d.'s (except the e.s.d. in the dihedral angle between two l.s. planes) are estimated using the full covariance matrix. The cell e.s.d.'s are taken into account individually in the estimation of e.s.d.'s in distances, angles and torsion angles; correlations between e.s.d.'s in cell parameters are only used when they are defined by crystal symmetry. An approximate (isotropic) treatment of cell e.s.d.'s is used for estimating e.s.d.'s involving l.s. planes.
Refinement. Refinement of *F*^2^ against ALL reflections. The weighted *R*-factor *wR* and goodness of fit *S* are based on *F*^2^, conventional *R*-factors *R* are based on *F*, with *F* set to zero for negative *F*^2^. The threshold expression of *F*^2^ > σ(*F*^2^) is used only for calculating *R*-factors(gt) *etc*. and is not relevant to the choice of reflections for refinement. *R*-factors based on *F*^2^ are statistically about twice as large as those based on *F*, and *R*- factors based on ALL data will be even larger.

### Fractional atomic coordinates and isotropic or equivalent isotropic displacement parameters (Å^2^)

**Table:**

	*x*	*y*	*z*	*U*_iso_*/*U*_eq_	
N1	0.7058 (3)	−0.0828 (3)	0.08266 (19)	0.0465 (7)	
N2	0.2416 (3)	0.2204 (3)	0.49248 (18)	0.0434 (7)	
O1	0.7764 (3)	−0.1760 (3)	0.00028 (16)	0.0581 (7)	
H1	0.8534	−0.1382	−0.0402	0.087*	
O2	0.0132 (3)	0.2394 (3)	0.66543 (16)	0.0557 (6)	
H2	0.0742	0.1976	0.6145	0.084*	
O3	−0.1419 (3)	0.3878 (3)	0.83000 (16)	0.0588 (7)	
O4	0.0191 (3)	0.9607 (3)	0.87286 (16)	0.0640 (7)	
H4A	−0.0095	1.0490	0.8152	0.077*	
H4B	0.0937	0.9763	0.9057	0.077*	
C1	0.5205 (4)	−0.2745 (4)	0.1335 (3)	0.0639 (10)	
H1A	0.4568	−0.2300	0.0665	0.096*	
H1B	0.4444	−0.3084	0.1984	0.096*	
H1C	0.6208	−0.3762	0.1267	0.096*	
C2	0.5816 (4)	−0.1332 (4)	0.1469 (2)	0.0410 (8)	
C3	0.4953 (4)	−0.0402 (4)	0.2358 (2)	0.0398 (8)	
C4	0.5415 (4)	0.0994 (5)	0.2463 (3)	0.0587 (10)	
H4	0.6288	0.1356	0.1957	0.070*	
C5	0.4632 (4)	0.1858 (4)	0.3285 (3)	0.0582 (10)	
H5	0.4994	0.2778	0.3329	0.070*	
C6	0.3319 (4)	0.1393 (4)	0.4047 (2)	0.0422 (8)	
C7	0.2842 (4)	0.0012 (4)	0.3959 (2)	0.0517 (9)	
H7	0.1968	−0.0342	0.4468	0.062*	
C8	0.3632 (4)	−0.0859 (4)	0.3131 (2)	0.0517 (9)	
H8	0.3268	−0.1780	0.3091	0.062*	
C9	0.2666 (4)	0.3593 (4)	0.5021 (2)	0.0464 (8)	
H9	0.3440	0.4082	0.4491	0.056*	
C10	0.1800 (4)	0.4421 (4)	0.5912 (2)	0.0429 (8)	
C11	0.0578 (4)	0.3787 (4)	0.6698 (2)	0.0408 (8)	
C12	−0.0257 (4)	0.4610 (4)	0.7574 (2)	0.0432 (8)	
C13	0.0148 (4)	0.6037 (4)	0.7638 (2)	0.0491 (8)	
H13	−0.0385	0.6574	0.8220	0.059*	
C14	0.1346 (4)	0.6693 (4)	0.6846 (3)	0.0589 (10)	
H14	0.1598	0.7674	0.6894	0.071*	
C15	0.2161 (4)	0.5905 (4)	0.5991 (3)	0.0561 (9)	
H15	0.2958	0.6359	0.5461	0.067*	
C16	−0.2143 (4)	0.4549 (5)	0.9261 (2)	0.0670 (11)	
H16A	−0.1202	0.4480	0.9665	0.100*	
H16B	−0.2844	0.3853	0.9749	0.100*	
H16C	−0.2872	0.5766	0.9017	0.100*	

### Atomic displacement parameters (Å^2^)

**Table:**

	*U*^11^	*U*^22^	*U*^33^	*U*^12^	*U*^13^	*U*^23^
N1	0.0498 (17)	0.0535 (18)	0.0406 (14)	−0.0166 (14)	0.0027 (13)	−0.0219 (14)
N2	0.0459 (17)	0.0426 (17)	0.0378 (14)	−0.0102 (13)	0.0004 (12)	−0.0117 (13)
O1	0.0661 (16)	0.0676 (17)	0.0528 (13)	−0.0311 (13)	0.0165 (11)	−0.0330 (12)
O2	0.0715 (16)	0.0571 (15)	0.0516 (13)	−0.0332 (13)	0.0125 (11)	−0.0271 (12)
O3	0.0683 (16)	0.0705 (17)	0.0538 (14)	−0.0367 (13)	0.0183 (12)	−0.0349 (13)
O4	0.0766 (17)	0.0707 (17)	0.0532 (13)	−0.0408 (14)	0.0019 (12)	−0.0119 (12)
C1	0.068 (2)	0.067 (3)	0.071 (2)	−0.032 (2)	0.0146 (19)	−0.035 (2)
C2	0.043 (2)	0.043 (2)	0.0394 (18)	−0.0172 (17)	−0.0020 (15)	−0.0110 (15)
C3	0.041 (2)	0.041 (2)	0.0370 (17)	−0.0125 (16)	−0.0042 (14)	−0.0102 (15)
C4	0.061 (2)	0.073 (3)	0.057 (2)	−0.039 (2)	0.0220 (18)	−0.031 (2)
C5	0.054 (2)	0.074 (3)	0.065 (2)	−0.035 (2)	0.0180 (18)	−0.040 (2)
C6	0.044 (2)	0.043 (2)	0.0387 (18)	−0.0133 (17)	−0.0002 (15)	−0.0108 (16)
C7	0.055 (2)	0.048 (2)	0.0474 (19)	−0.0219 (18)	0.0168 (16)	−0.0121 (17)
C8	0.054 (2)	0.047 (2)	0.056 (2)	−0.0234 (18)	0.0096 (17)	−0.0158 (18)
C9	0.044 (2)	0.042 (2)	0.0442 (18)	−0.0100 (17)	0.0051 (15)	−0.0078 (16)
C10	0.040 (2)	0.043 (2)	0.0417 (18)	−0.0108 (17)	−0.0001 (15)	−0.0104 (16)
C11	0.047 (2)	0.038 (2)	0.0411 (18)	−0.0146 (16)	−0.0057 (15)	−0.0128 (15)
C12	0.041 (2)	0.045 (2)	0.0449 (18)	−0.0146 (17)	−0.0033 (15)	−0.0132 (16)
C13	0.052 (2)	0.047 (2)	0.053 (2)	−0.0151 (18)	−0.0034 (17)	−0.0207 (17)
C14	0.065 (2)	0.049 (2)	0.072 (2)	−0.024 (2)	−0.002 (2)	−0.025 (2)
C15	0.060 (2)	0.044 (2)	0.064 (2)	−0.0242 (19)	0.0098 (18)	−0.0135 (18)
C16	0.075 (3)	0.086 (3)	0.052 (2)	−0.034 (2)	0.0175 (18)	−0.036 (2)

### Geometric parameters (Å, °)

**Table:**

N1—C2	1.281 (3)	C5—C6	1.377 (4)
N1—O1	1.398 (3)	C5—H5	0.9300
N2—C9	1.285 (3)	C6—C7	1.375 (4)
N2—C6	1.418 (3)	C7—C8	1.378 (4)
O1—H1	0.8200	C7—H7	0.9300
O2—C11	1.349 (3)	C8—H8	0.9300
O2—H2	0.8200	C9—C10	1.434 (4)
O3—C12	1.357 (3)	C9—H9	0.9300
O3—C16	1.422 (3)	C10—C11	1.388 (4)
O4—H4A	0.8500	C10—C15	1.400 (4)
O4—H4B	0.8500	C11—C12	1.412 (4)
C1—C2	1.489 (4)	C12—C13	1.367 (4)
C1—H1A	0.9600	C13—C14	1.386 (4)
C1—H1B	0.9600	C13—H13	0.9300
C1—H1C	0.9600	C14—C15	1.371 (4)
C2—C3	1.480 (4)	C14—H14	0.9300
C3—C8	1.386 (4)	C15—H15	0.9300
C3—C4	1.388 (4)	C16—H16A	0.9600
C4—C5	1.369 (4)	C16—H16B	0.9600
C4—H4	0.9300	C16—H16C	0.9600
			
C2—N1—O1	112.3 (2)	C7—C8—C3	121.9 (3)
C9—N2—C6	121.4 (3)	C7—C8—H8	119.1
N1—O1—H1	109.5	C3—C8—H8	119.1
C11—O2—H2	109.5	N2—C9—C10	122.7 (3)
C12—O3—C16	117.0 (2)	N2—C9—H9	118.6
H4A—O4—H4B	107.7	C10—C9—H9	118.6
C2—C1—H1A	109.5	C11—C10—C15	119.0 (3)
C2—C1—H1B	109.5	C11—C10—C9	120.9 (3)
H1A—C1—H1B	109.5	C15—C10—C9	120.1 (3)
C2—C1—H1C	109.5	O2—C11—C10	122.1 (3)
H1A—C1—H1C	109.5	O2—C11—C12	117.6 (3)
H1B—C1—H1C	109.5	C10—C11—C12	120.2 (3)
N1—C2—C3	116.7 (3)	O3—C12—C13	125.3 (3)
N1—C2—C1	123.0 (3)	O3—C12—C11	115.4 (3)
C3—C2—C1	120.3 (3)	C13—C12—C11	119.3 (3)
C8—C3—C4	115.9 (3)	C12—C13—C14	120.7 (3)
C8—C3—C2	122.4 (3)	C12—C13—H13	119.6
C4—C3—C2	121.8 (3)	C14—C13—H13	119.6
C5—C4—C3	122.3 (3)	C15—C14—C13	120.4 (3)
C5—C4—H4	118.9	C15—C14—H14	119.8
C3—C4—H4	118.9	C13—C14—H14	119.8
C4—C5—C6	121.3 (3)	C14—C15—C10	120.3 (3)
C4—C5—H5	119.4	C14—C15—H15	119.8
C6—C5—H5	119.4	C10—C15—H15	119.8
C7—C6—C5	117.3 (3)	O3—C16—H16A	109.5
C7—C6—N2	116.8 (3)	O3—C16—H16B	109.5
C5—C6—N2	125.9 (3)	H16A—C16—H16B	109.5
C6—C7—C8	121.3 (3)	O3—C16—H16C	109.5
C6—C7—H7	119.3	H16A—C16—H16C	109.5
C8—C7—H7	119.3	H16B—C16—H16C	109.5
			
O1—N1—C2—C3	−178.9 (2)	N2—C9—C10—C11	−2.6 (4)
O1—N1—C2—C1	−0.4 (4)	N2—C9—C10—C15	178.3 (3)
N1—C2—C3—C8	−177.9 (3)	C15—C10—C11—O2	178.2 (3)
C1—C2—C3—C8	3.6 (4)	C9—C10—C11—O2	−0.9 (4)
N1—C2—C3—C4	2.5 (4)	C15—C10—C11—C12	−1.2 (4)
C1—C2—C3—C4	−176.0 (3)	C9—C10—C11—C12	179.7 (3)
C8—C3—C4—C5	0.7 (5)	C16—O3—C12—C13	−6.2 (4)
C2—C3—C4—C5	−179.7 (3)	C16—O3—C12—C11	173.5 (2)
C3—C4—C5—C6	−0.8 (5)	O2—C11—C12—O3	1.0 (4)
C4—C5—C6—C7	0.8 (5)	C10—C11—C12—O3	−179.6 (2)
C4—C5—C6—N2	−179.6 (3)	O2—C11—C12—C13	−179.3 (3)
C9—N2—C6—C7	−174.4 (3)	C10—C11—C12—C13	0.1 (4)
C9—N2—C6—C5	6.0 (5)	O3—C12—C13—C14	−179.4 (3)
C5—C6—C7—C8	−0.7 (5)	C11—C12—C13—C14	0.9 (5)
N2—C6—C7—C8	179.6 (3)	C12—C13—C14—C15	−0.8 (5)
C6—C7—C8—C3	0.7 (5)	C13—C14—C15—C10	−0.4 (5)
C4—C3—C8—C7	−0.6 (5)	C11—C10—C15—C14	1.4 (5)
C2—C3—C8—C7	179.7 (3)	C9—C10—C15—C14	−179.5 (3)
C6—N2—C9—C10	−178.7 (2)		

### Hydrogen-bond geometry (Å, °)

**Table:**

*D*—H···*A*	*D*—H	H···*A*	*D*···*A*	*D*—H···*A*
O1—H1···O4^i^	0.82	1.84	2.656 (3)	176
O2—H2···N2	0.82	1.86	2.589 (3)	147
O4—H4A···O2^ii^	0.85	2.07	2.885 (3)	161
O4—H4B···N1^iii^	0.85	2.15	2.945 (3)	156

Symmetry codes: (i) *x*+1, *y*−1, *z*−1;  (ii) *x*, *y*+1, *z*; (iii) −*x*+1, −*y*+1, −*z*+1.
